# Erratum for Wan et al., “Biological characteristics of mechanosensitive channels MscS and MscL in *Actinobacillus pleuropneumoniae*”

**DOI:** 10.1128/jb.00237-25

**Published:** 2025-09-02

**Authors:** Jiajia Wan, Lu Dai, Huasong Xiao, Wendie Zhang, Rui Zhang, Tingting Xie, Yizhen Jia, Xuejun Gao, Jing Huang, Feng Liu

## ERRATUM

Volume 206, no. 3, e00429-23, 2024, https://doi.org/10.1128/jb.00429-23. Page 5, Fig. 2D: “42°C” should read “0.3 M Na^+^.”

Page 5, Legend for Fig. 2, “0.3 mM Na^+^” should read “0.3 M Na^+^.”

